# Prescription Pattern of Antihypertensive Agents in T2DM Patients Visiting Tertiary Care Centre in North India

**DOI:** 10.1155/2012/520915

**Published:** 2012-12-18

**Authors:** Ethiraj Dhanaraj, Amit Raval, Rajbharan Yadav, Anil Bhansali, Pramil Tiwari

**Affiliations:** ^1^Department of Pharmacy Practice, National Institute of Pharmaceutical Education and Research (NIPER), Punjab, S.A.S. Nagar 160062, India; ^2^Department of Pharmaceutical Systems and Policy, School of Pharmacy, West Virginia University, 1 Medical Center Drive, Morgantown, WV 26505, USA; ^3^Department of Endocrinology, Post Graduate Institute of Medical Education and Research, Chandigarh 160012, India

## Abstract

*Background*. Hypertension management is of a paramount importance in diabetic patients for cardiovascular risk reduction. *Aim*. To evaluate prescribing pattern of antihypertensive in T2DM (type 2 diabetes) patients and compare with existing recent guidelines. *Methods*. A cross-sectional study involving evaluation of all T2DM patients referred to endocrinology unit at tertiary care centre for hypertension, comorbid complications, and recording prescription. Utilization of 5 different antihypertensive drug classes was compared for all patients receiving 1, 2, 3, 4, or more drugs. Logistical regression was used to assess likelihood of prescription of drugs and/or therapy for specific conditions mentioned in the guidelines. *Results*. Out of 1358, T2DM enrolled patients 1186 (87%) had hypertension (males 52%, females 48%). The median duration (IQ) of hypertension diabetics was 4 (1–10) years. A total of 25% patients had controlled BP and 75% with uncontrolled blood pressure (13% isolated systolic hypertension, 6% isolated diastolic hypertension, and 55% both elevated). Overall, ACE inhibitors (ACEIs) were prescribed the highest (59%) followed by angiotensin receptor blockers (ARBs) (52%), calcium channel blockers (CCBs) (29%), diuretics (27%), and beta-blockers (14%). Overall, 55% of T2DM patients were on polytherapy, 41% on monotherapy, and 4% had no antihypertensive treatment. Polytherapy was more predominant with age, duration of diabetes, duration of hypertension, and comorbid complications. *Conclusion*. Although prescribing pattern of antihypertensive showed adherence to existing evidence-based guidelines, higher proportion of uncontrolled hypertensive patients was found.

## 1. Introduction

Diabetes and hypertension are the major burden of global Health. The World Health Organization projected that 300 million people will suffer from diabetes and 1.5 billion [1] from hypertension by 2025 [2]. According to the Diabetes Atlas 2006 published by the International Diabetes Federation, the number of people with diabetes in India currently around 40.9 million, is expected to rise to 69.9 million by 2025 unless urgent preventive steps are taken [3]. The incidence of hypertension in patients with T2DM is approximately two-fold higher than in age-matched subjects without the disease [4].

Hypertension has been identified as a major risk factor not only for the development of diabetes but also for the development of micro and macro vascular complications, that is, neuropathy, nephropathy, retinopathy, coronary artery disease (CAD), stroke, peripheral vascular disease (PVD) in diabetic patients. It has been evident from Framingham heart study, UKPDS (United Kingdom Prospective Study-39), Hypertension Optimal Treatment (HOT), Systolic Hypertension in the Elderly Programme (SHEP), Systolic hypertension in Europe (SYST-Eur), Hypertension in the Very Elderly Trial (HYVET-Pilot) that reduction in either isolated systolic or systolic-diastolic hypertension significantly reduces the risk of micro and macro vascular complications and cardiovascular (CV) death or diabetes-related death [5–11]. Lowering blood pressure (BP) in patients with diabetes mellitus is more cost effective than tight blood glucose control, and beneficial results are apparent earlier [8]. Therefore, all of the hypertension management guidelines, that is, Seventh Report of Joint National Committee on Prevention, Detection, Evaluation, and Treatment of High Blood Pressure-2003 (JNC-7), American Diabetes association (ADA) 2010, European Society of Hypertension (ESH) [11], WHO guideline [8, 10, 12] focused aggressively on blood pressure (BP) control in diabetic patient to below 130–135/80–85 mmHg [8, 9, 12].

Given the importance of the blood pressure control in diabetic patients, there has been much needed data on achievement of target control in real world diabetic hypertensive patients; however, the data on prescribing pattern as well as blood pressure control is scare especially in Asian-Indian subcontinent. Thus, this study was conducted to assess blood pressure control and the prescribing patterns of antihypertensive in hypertensive diabetic patients in real world clinical settings. Further, the study also assessed adherence of prescribing pattern with existing guidelines.

## 2. Subject and Methods 

This cross-sectional study was carried out from June 2008 to May 2009 at the Nehru Hospital, Postgraduate Institute of Medical Education and Research, Chandigarh. Patients were confirmed by physician diagnosed T2DM patients, were further examined consecutively for social, demographical, and clinical variables. Informed and written consent was obtained from all the participants after full explanation of the procedure. The study protocol was approved by the Institute's Ethics Committee.

### 2.1. Anthropometric Characteristics

Standing body height (to the nearest 0.5 cm) was measured with a commercial stadiometer. A digital scale, with an accuracy of ±100 g, was used to measure body weight (BW). The waist circumference (WC) was measured in a horizontal plane, midway between the inferior margin of the ribs and the superior border of the iliac crest. Hip circumference (HC) was measured at the fullest point around the buttocks with a metallic tape. The measurements were taken thrice and the mean was taken in all cases. WC (cm) was divided by HC (cm) to calculate waist to hip ratio (WHR). Body mass index (BMI) (kg/m^2^) was calculated by dividing weight (in kilograms) by the square of height (in meters), as a measure of total adiposity. Percent body fat (%BF) was evaluated by impedance plethysmography (bioelectrical impedance meter (Omron BF 302, Tokyo)). 

### 2.2. Blood Pressure Measurement

Systolic and diastolic blood pressure (SBP & DBP) were measured twice at an interval of 3 min in the sitting position after a 15 min rest, and the mean was taken. Further, hypertension conformation was based on any of following criteria: at least two outpatient visit diagnosis of hypertension; at least 1 prescription of antihypertensive drug plus at least 1 outpatient diagnosis of hypertension; At least 2 elevated BP measurements plus one outpatients diagnosis of hypertension; at least two elevated blood pressure measurement. According to JNC-7 report (Joint National Committee on Prevention, Detection, Evaluation and Treatment of High Blood Pressure), elevated blood pressure was defined as higher than or equal to 130/80 mmHg.

### 2.3. Biochemical and Clinical Parameters

Blood samples (3 mL) were drawn after 8–12 h overnight fasting for the measurement of lipid profile (total cholesterol, high density lipoprotein (HDL) cholesterol, and triglycerides) and fasting plasma glucose levels. Plasma glucose was measured using the glucose oxidaseperoxidase method [13], serum total cholesterol [14], and triglycerides [15] by standard enzymatic procedures and HDL cholesterol [16] by direct assay method. Uncontrolled hyperglycemia was defined as HbA1c (Glycosylated heamoglobin A1c) >7% or FPG (Fasting Plasma Glucose) >110 mg/dL or PPG (PostPrandial Glucose) >140 mg/dL.

Neuropathy was evaluated by history of numbness, paraesthesias, tingling sensation, burning sensation, and confirmed by touch sensation using 10 gm monofilament, vibration sense by biothesiometer (VPT at great toe >25 mV were considered significant) and ankle reflex. Incipient nephropathy was diagnosed by Micral test and it was presumed to be present if any two readings out of three of urinary albumin and creatinine ratio were ranging from 30 to 300 *μ*g/mg. Clinical nephropathy was evaluated by the estimation of 24 hr urine protein, and it was present if urine proteins were more than 500 mg/total volume of urine. Ophthalmologist diagnosed retinopathy by detailed fundus examination and was classified according to Diabetic Retinopathy Study (DRS) and Early Treatment Diabetic Retinopathy Study (ETDRS) [17]. Coronary artery disease was diagnosed by history of angina or myocardial infarction or documented by previous treatment records. Interpretation of ECG was recorded as per Minnesota codes. Pathological Q wave (major Q wave abnormalities) in an ECG recording (Minnesota codes 1.1.1–1.2.7), ST segment depression (codes 4.1-4.2), T wave abnormalities (codes 5.1–5.4), and chest X-ray was done to assess cardiac size. Peripheral vascular disease (PVD) was diagnosed by definitive history of intermittent claudication or if one or more peripheral pulses were absent in both feet. The grading was done according to ankle brachial pressure index (ABPI) by Doppler study [Multi Duplex (R)-II (Huntleigh Diagnostics-UK)]. PVD was diagnosed when ankle brachial index was less than 0.9. 

All type 1 diabetic patient with hypertension, pregnant or breast feeding women, were excluded.

### 2.4. Statistical Analysis

Proportion of patients using 5 different classes of antihypertensive was calculated. For prescription pattern analysis, immunotherapy was considered as a single drug at any frequency of any antihypertensive class, while polytherapy was considered as a combination of two antihypertensive drugs from two different classes at any dose and frequency. Prescribing pattern with five different classes of antihypertensive were analyzed in blood pressure control and diabetic complications. Data were validated after double entry and then analyzed using the Statistical Package for Social Science (SPSS) (*Version *16 USA). Binary logistic regression model was used to examine association between predictor variables (as captured by data capturing form) and prescription of a particular class of drug. The main model consists of the following predictor variables: demographic variables, age, gender, obesity, clinical variables like diabetic complications, blood pressure and duration of disease, and so forth. Results are expressed as OR and 95% confidence intervals (CI). 

## 3. Results

Out of 1358 diabetic patients, 1186 (87%) patients were found to have hypertension. The average age (SD) of type diabetic hypertensive patients was 55.6 (10.1) years comprising 620 (52%) male and 566 (48%) female patients. The average duration of diabetes (DOD) (SD) was 9.6 (7.5) years and median duration of hypertension was 4 (1–10) years. A total of 224 (19%) was newly diagnosed to be hypertensive with average age 53 (11) and DOD of 7.4 (7). Male had higher DOD and DHTN to their counterpart female (*P* < 0.05). (See Table 1) Overall blood pressure control was achieved in 293 (24%) patients, 121 (25%) in patients on monotherapy, and 172 (26%) in patients receiving multidrug regimens. 

### 3.1. Most Frequently Used Antihypertensive Drugs

Overall, 46 (4%) patients were not on any pharmacotherapy, 485 (41%) patients on one drug (monotherapy), 655 (55%) on more than one drugs (polytherapy) (see Table 2). Among the patients with polytherapy, 423 (65%) patients were on two drugs, 195 (30%) on 3 drugs, and 37 (6%) were on combination of four or more drugs.

Most patients were receiving ACEI and/or ARB [1037 (88%)]. As individual drug class, ACEIs were utilized highest 627 (59%), followed by ARBs 620 (52%), CCBs 340(29%) diuretics 315 (27%) and BB 167 (14%) either as mono or combination therapy (see Table 2). However ARB was utilized higher than ACEIs among patients as polytherapy, where ARB use ranged from 60% to 97% and ACEIs use ranged from 57% to 78%. Patients on monotherapy were mostly receiving ACEIs/ARBs 403 (83%). Beta-blockers use ranged from 5% in patients on monotherapy to 65% in patients receiving four or more medications. 

In two drugs combination therapy, ACEIs plus ARBs were prescribed highest 89 (21%), followed by ARBs plus diuretics 76 (18%), ACEIs plus CCBs 72 (17%), and ARB plus CCBs 67 (16%). In three drug combinations, combination of ACEI plus ARB plus diuretics were highest with 58 (30%) followed by ARB plus CCB plus diuretics 41 (21%). In 4 or more drug combinations of ARB, ACEI, CCBS plus diuretics were commonly prescribed [13 (35%)] (see Table 3). A total of 25% patient had controlled BP, 75%with uncontrolled blood pressure (13% isolated systolic hypertension, 6% isolated diastolic hypertension, 55% both elevated).

### 3.2. Choice of Antihypertensive Drugs

Table 4 shows results of logistical regression analysis. In univariate analysis, age, duration of diabetes and duration of hypertension, BMI, waist, Hb_A1c_, neuropathy, nephropathy, retinopathy, CAD, and DF foot complications were taken as independent variable. Patients with >55 years were more likely to receive diuretics compared to patients with ≤55 years. (OR: 1.17, 95% CI 1.03–1.35). Prescription of any hypertensive drugs were not associated with gender or duration of diabetes, however polytherapy was more predominant in elderly (*P* < 0.05) and was positively associated with duration of diabetes (OR: 2.172, 95% CI 1.462–3.226) and duration of hypertension (OR: 2.11, 95% CI 1.35–3.12). ARBs, calcium channel blocker, and diuretics were more likely to be prescribed when considered as mono or combination therapy (OR: 1.61, 95% CI 1.24–2.08, 1.27, 95% CI 1.05–1.51, 2.05, 95% CI 1.55–2.74, resp.). Prescription pattern of any antihypertensive drugs did not significantly associate with neuropathy. The prevalence of nephropathy was 480 (41%). The significant difference was observed in patients with mono and poly therapy with nephropathy (*P* < 0.05). ARBs and ACEIs were the drug of choice for nephropathy. ARB and diuretics were more likely to be prescribed in nephropathy (OR: 1.43, 95% CI 1.12–1.83, 1.42, 95% CI 1.08–1.88 resp.). In addition patients with elevated serum creatinin level (>1.2 mg/dL) ACE were less likely to be prescribed (OR: 0.84, 95% CI 0.71–0.99). 

The prevalence of CAD was found to be 13% (159). Patients with CAD were more likely to be prescribed polytherapy (*P* < 0.05). Patients with CAD were likely to get ACEI/ARB with diuretics (OR: 2.39, 95%CI 1.25–4.59). Beta-blockers were more likely to prescribe both in mono and polytherapy (OR: 2.52, 95% CI 1.44–4.41). 

## 4. Discussion

In present study, ACEI and ARBs were more commonly prescribed drugs, followed by calcium channel blocker, diuretics and beta-blockers irrespective of mono or poly therapy. Majority of patients were on polytherapy in the present study. 

Blood pressure lowering treatment trialists (BPLTT) collaborations meta analysed evidence of clinical trials on treatment of hypertension, which showed significant benefits from a more intense blood pressure reduction in stroke and major cardiovascular events. Aggressive antihypertensive treatment, although difficult to achieve, resulted in substantial reductions of left ventricular mass (LVM) index and arterial stiffness in relatively uncomplicated hypertensive T2DM patients [18, 19]. 

The choice of antihypertensive drug should be determined by the drug's capacity to lower pressure, to protect the diabetic patient's kidney from ongoing injury and cardiovascular complications. Antihypertensive and lipid-lowering teatment to prevent heart attack trial (ALLHAT) compared metabolic, cardiovascular, and renal outcomes in individuals assigned to initial hypertension treatment with a thiazide-like diuretic (chlorthalidone), a calcium channel blocker (CCB; amlodipine), or an ACE inhibitor (lisinopril) in nondiabetic individuals with or without metabolic syndrome. It showed despite a less favorable metabolic profile, thiazide-like diuretic initial therapy for hypertension offers similar, and in some instances possibly superior, CVD outcomes in older hypertensive adults with metabolic syndrome, as compared with treatment with CCBs and ACE inhibitors strategies based on renin-angiotensin system inhibitors were not clearly superior to conventional (i.e., diuretic-based) strategies [20]. Furthermore, ACEIs showed to reduced incidence of coronary heart disease compared to diuretics (ALLAHAT) and reducing cardiovascular event compare to CCB [21–23], but heart failure and stroke were lower in diuretics.

ACEIs have shown a specifically beneficial effect in microvascular disease in kidney. It is mainly due to decreasing capillary perfusion, reducing transcapillary leakage of albumin, and in long run decrease damage to both capillaries and arteries [24]. It has been shown titrated dose of ACEIs in nephropathy according to level of blood pressure has more significant complications. In addition to ACEIs, ARBs have shown benefits not only in nephropathy, heart failure protection but also in reduceding incidence of hyperkalemia and dry cough [19]. 

The UKPDS showed the beneficial effects of the ACE inhibitor captopril on diabetes-related mortality and microvascular and cardiovascular complications in patients with type 2 diabetes [25], ACE inhibitors are also effective in decreasing cardiovascular mortality and morbidity inpatients with congestive heart failure and postmyocardial infarction [26, 27]. Finally, the use of the ACE inhibitor ramipril in the heart outcomes prevention evaluation (HOPE) trial resulted in areduction in all-cause and cardiovascular mortality as well as cardiovascular events, including myocardial infarction and stroke [28]. Reductions in cardiovascular end points were seen regardless of improvements in blood pressure, suggesting that ACE inhibitors have benefits that are independent of their antihypertensive effects [26–28].

European guidelines utilized cost-effective treatment of antihypertensive treatments based on sound economic model ling. Guidelines suggested that strategy based CCBs are the most cost effective and BB were least cost effective [29–31]. In present study cost-effectively of treatment was in accordance with their treatment guidelines.

### 4.1. Drug Classes, Monotherapy versus Polytherapy

ESH suggests ARBs should be a regular component of combination treatment and preferred one when monotherapy alone in diabetics [30]. In addition, initial monotherapy ACE inhibitors may be superior to dihydropyridine calcium channel blockers in reducing cardiovascular events [32–34]. In addition an advantage on cardiovascular outcomes of initial therapy with low-dose thiazide diuretics [26, 35].

In the present study with the prescription of antihypertensive medication was consistent with guidelines as significantly high use of ACEI with low dose of diuretics in high risk groups for cardiovascular events. Initial choice of monotherapy was mostly ACEIs or ARBs (76%). 

Use of multiple drugs in combinations is being increasingly recognized as critical to control hypertension in patients with diabetes. Several large clinical trials demonstrated that most patients with hypertension could achieve and sustain adequate blood pressure control only with the use of multiple antihypertensive drugs [31, 36]. A large proportion of treated patients (59%) were being prescribed multidrug regimens. In addition, it was intensified with increasing age, duration of diabetes, duration of hypertension or if complications or comorbidities were present, this was in consistency with treatment pattern of the evidence-based guidelines.

Dual blockade of the reninangiotensin system using ARBs and ACEIs (the Candesartan and Lisinopril Microalbuminuria [CALM] study) found that the combination of both agents reduced blood pressure and urinary albumin levels to a greater extent than either medication alone [8].

### 4.2. Antihypertensive Use with Respect to Isolated Hypertension

In diabetic patients >65 years of age with isolated systolic hypertension (i.e., >140 mmHg systolic and <80 mmHg diastolic blood pressure), pharmacological treatment should be initiated. Earlier recommendations to treat a systolic blood pressure <160 mmHg have been reduced based on the increased cardiovascular risk of these patients and the results of the SHEP study, in which a systolic blood pressure of 144 mmHg was achieved. Combinations of agents are often required [37]. When drug therapy is intensified, patients should be monitored carefully for adverse effects, such as orthostatic hypotension.

### 4.3. Isolated Systolic Hypertension

Diuretics were more utilized in combinations with other antihypertensive drugs in elderly compare to nonelderly patients A meta analysis has suggested that in elderly, diuretics have more pronounced preventing effects on cardiovascular mortality. In trials of isolated systolic hypertension first line comprised diuretics or calcium channel blocker [8, 38, 39]. Subgroup analysis of trials on isolated hypertension shown efficacy of ARBs [40, 41]. 

### 4.4. Antihypertensive Drug Use with Respect to Diabetic Complications

It has been shown that ACEIs as well as conventional antihypertensive delays progression of nephropathy [42]. Available evidence show that the presence of microalbunuria is not only early marker of renal diasease but also indiactor of increased cardiovascular risk. Hence, all recent guidelines [11, 29–31, 43] accentuate on use of ACEI as a first choice for diabetic hypertension.

In present study, among 219 overt nephropathy patients 120 were on ARBs (OR: 1.693, 95% CI: 1.168–2.463) and 116 on ACEIs (OR: 1.094, 95% CI: 0.756–1.582). A meta analysis has shown ACEIs to be effective for the primary prevention of kidney disease in diabetes and with ACEIs, a RR reduction of 42% has been demonstrated (95% CI 16% to 60%). ACEIs alone or with low dose of diuretics delay end stage renal disease or prevent microalbunuria or proteinuria [11, 41]. ADA guidelines recommends that proteinuric patients, especially those with diabetes mellitus, need aggressive BP control and use of ACEIs and/or ARBs [11]. In addition, in those with type 2 diabetes, hypertension, macroalbuminuria (300 mg/day), and renal insufficiency an ARB should be strongly considered [11]. 

 ACE and ARB were found to be major category for two-drug combination use. Recent studies found that combination of ACE and ARB compared with ACE inhibitors alone, was associated with significant increases in renal dysfunction and hyperkalemia, poorly tolerated, patients less adhere to combination therapy due to adverse effect [10]. The current diabetes guidelines did not clearly avoid mentioning use of this combination, instead suggested use of ARB for diabetic nephropathy in addition to ACE [44]. Our studies results showed that negative outcomes of ACE plus ARB use in those studies did not influence the prescribing patterns.

### 4.5. Cardiovascular Complications

In patients with CHF, ARBs were superior to calcium channel blocker for reducing heart failure [43, 45], hence ADA recommends it as first line drug.

Treatment with beta blocker has a protective effect on cardiovascular mortality after myocardial infraction, as this is a major cause of disease. So, prophylaxis of BBs are advisable with high risk patients. In present study, it was significantly higher proportion were on beta blocker with coronary artery disease [25].

The rate of successful blood pressure control was 26% compared higher hypertensive patients receiving treatment, and despite the inadequacy of monotherapy to control blood pressure, many of the patients received this treatment regimen. We found overall BP control (<130/85) to be 32%. Obviously, BP control is multifactorial, with factors such as age, comorbidity, and patient adherence to medication regimens affecting this outcome, and our study does not attempt to examine all other parameters like adherence to the method.

### 4.6. Conclusion

We found that however prescribing patterns was consistent with the evidence-based guideline, only one fourth of diabetic patient had blood pressure within the target. However, the result is encouraging as it is better compared to previous report from the same hospital which suggested only 11% T2DM patient have hypertension under control.

## Figures and Tables

**Table 1:** Demographic and clinical parameters of hypertensive T2DM patients.

Characteristics	Total [1186 (100)]	Men [620 (52)]	Women [566 (48)]	*P* value
Age (Yrs)	55.6 ± 10.1	56.2 ± 10.1	54.9 ± 10	0.042
BMI (Kg/m^2^)	26.5 ± 9.6	26.8 ± 12.1	26.3 ± 4.6	0.759
DOD (Yrs)	9.6 ± 7.5	10.3 ± 8.1	8.9 ± 6.8	0.0015
DHTN (Yrs)	4 (1–10)	4 (1–9)	4 (1–10)	0.001
Waist Circumference (cm)	93.9 ± 11.6	93.7 ± 12	94.2 ± 11.2	0.502
SBP (mmHg)	141.2 ± 18.2	140.7 ± 18.2	142.2 ± 18.2	0.103
DBP (mmHg)	85.7 ± 10.8	86.6 ± 11	87.2 ± 10.6	0.249
FPG (mg/dL)	144.2 ± 58.5	145.7 ± 60.4	142.6 ± 56.4	0.477
PPG (mg/dL)	204.3 ± 77.8	208.9 ± 81.7	199.3 ± 72.9	0.057
Hb_A1c_ (%)	9.1 ± 15.6	9.2 ± 15.7	8.9 ± 15.5	0.045
Scr (mg/dL)	1.18 ± 0.87	1.12 ± 0.7	1.25 ± 1.04	0.121
TCh (mg/dL)	177.7 ± 51.9	179.7 ± 53.4	175.4 ± 50.2	0.04
LDL (mg/dL)	84.4 ± 42.5	85.6 ± 45.1	83.2 ± 39.5	0.079
HDL (mg/dL)	67.2 ± 39.1	68.4 ± 39.8	65.6 ± 38.5	0.521
TG (mg/dL)	157.6 ± 76.9	161.5 ± 78.7	153.1 ± 74.9	0.074

**Table 2:** Pattern of Antihypertensive among patients with single or multiple drugs hypertension.

Drug Classes *n* (%)	Overall	Monotherapy	Polytherapy			
			Overall	2 Drugs	3 Drugs	≥4 Drugs
Antihypertensives	1186 (100)	485 (41)	655 (55)	423 (36)	195 (16)	37 (3)
ACEIs	697 (59)	229 (47)	398 (61)	241 (57)	128 (66)	29 (78)
ARBs	620 (52)	174 (36)	446 (68)	253 (60)	157 (81)	36 (97)
BBs	167 (14)	23 (5)	144 (22)	65 (15)	55 (28)	24 (65)
CCBs	340 (29)	58 (12)	282 (43)	150 (35)	98 (50)	34 (92)
Diuretics	315 (27)	1 (0.2)	314 (48)	137 (32)	147 (75)	30 (81)

Note: Percentages (%) for individuals drug class in the second row are column %.

**Table 3:** Prescribing pattern in patients with controlled versus uncontrolled hypertension.

Drug Class *n* (%)	Overall [1185 (100)]	Controlled BP [293 (25)]	Uncontrolled BP [893 (75%)]		
			SBP[152 (13)]	DBP [81 (6)]	Both [660 (56)]
No drugs	46 (4)	—	—	6 (7%)	40 (7%)
Monotherapy	485 (41)	121 (25)	56 (12)	29 (6)	279 (33)
ACEI	229 (47)	60 (50)	25 (16)	14 (19)	130 (47)
ARB	174 (36)	44 (36)	16 (11)	13 (17)	101 (36)
BB	23 (5)	6 (5)	4 (3)	1 (1)	12 (4)
CCB	58 (12)	11 (9)	11 (7)	1 (1)	35 (13)
DI	1 (0.4)	—	—	—	1 (0.4)
Polytherapy	655 (65)	172 (26)	96 (15)	46 (7)	341 (33)
2 Drugs combination	423 (65)	110 (64)	66 (69)	33 (72)	214 (62)
ACEI + ARB	89 (21)	28 (25)	12 (18)	9 (27)	40 (19)
ACEI + BB	27 (6)	11 (10)	3 (5)	2 (6)	11 (5)
ACEI + CCB	72 (17)	18 (16)	16 (24)	6 (18)	32 (15)
ACEI + DI	53 (13)	17 (15)	6 (9)	2 (6)	28 (13)
ARB + BB	21 (5)	6 (5)	4 (6)	3 (9)	8 (4)
ARB + CCB	67 (16)	12 (11)	9 (14)	6 (18)	40 (19)
ARB + DI	76 (18)	12 (11)	11 (17)	3 (9)	50 (23)
BB + CCB	10 (2)	3 (3)	3 (5)	2 (6)	2 (1)
BB + DI	7 (2)	3 (3)	2 (3)	—	2 (1)
CCB + DI	1 (0.3)	—	—	—	1 (0.5)
3 Drugs combinations	195 (30)	51 (30)	29 (30)	12 (26)	103 (30)
ACEI + ARB + BB	14 (7)	5 (10)	2 (7)	1 (8)	6 (6)
ACEI + ARB + CCB	21 (11)	4 (8)	0 (0)	1 (8)	16 (16)
ACEI + ARB + DI	58 (30)	15 (29)	12 (41)	5 (42)	26 (25)
ACEI + BB + CCB	7 (4)	4 (8)	—	1 (8)	2 (2)
ACEI + BB + DI	8 (4)	3 (6)	1 (3)	—	4 (4)
ACEI + CCB + DI	20 (10)	5 (10)	2 (7)	4 (33)	9 (9)
ARB + BB + CCB	6 (3)	1 (2)	3 (10)	—	2 (2)
ARB + BB + DI	17 (9)	3 (6)	3 (10)	—	11 (112)
ARB + CCB + DI	41 (21)	8 (10)	6 (21)	—	27 (26)
BB + CCB + DI	3 (2)	3 (6)	—	—	—
4 or more drugs	37 (6)	11 (6)	1 (1)	1 (2)	24 (7)
ACEI + ARB + CCB + DI	13 (35)	4 (36)	1 (100)	1 (100)	7 (29)
ACEI + ARB + BB + CCB	7 (19)	4 (36)	—	—	3 (13)
ACEI + ARB + BB + DI	3 (8)	1 (9)	—	—	2 (8)
ACEI + BB + CCB + DI	1 (3)	1 (9)	—	—	—
ARB + BB + CCB + DI	8 (22)	1 (9)	—	—	7 (29)
ACEI + ARB + BB + CCB + DI	5 (14)	—	—	—	5 (21)

**Table 4:** Multiple logistical regression showing variables associated with prescribing of antihypertensive drugs.

Variables	ACEI		ARBs		BBs		CCBs		Diuretics	
	OR (95%CI)	*P* value	OR (95%CI)	*P* value	OR (95%CI)	*P* value	OR (95%CI)	*P* value	OR (95%CI)	*P* value
Age (≤55 versus >55 years)	1.00 (0.99–1.01)	0.986	1.21 (0.95–1.55)	0.12	0.99 (0.97–1.01)	0.394	1 (0.99–1.018)	0.482	**1.17** **(1.03–1.35) **	**0.019**
Sex (male versus female)	1.23 (0.96–1.57)	0.095	0.83 (0.65–1.06)	0.15	0.95 (0.66–1.36)	0.77	0.8 (0.61–1.05)	0.11	0.89 (0.68–1.18)	0.436
DDM (≤9 versus >9 years)	0.99 (0.77–1.28)	0.973	1.24 (0.96–1.59)	0.09	0.93 (0.81–1.06)	0.267	1.08 (0.82–1.43)	0.574	0.97 (0.72–1.28)	0.81
DHTN (≤5 versus >5 yrs)	1.07 (0.69–1.11)	0.57	**1.61** **(1.24–2.08)**	**<0.001**	1.26 (0.87–1.83)	0.219	**1.27** **(1.05–1.51)**	**0.04**	**2.05** **(1.55–2.74)**	**<0.001**
BMI* (≤5 versus >5 yrs)	1.14 (0.89–1.46)	0.3	1.03 (0.81–1.32)	0.806	1.00 (0.988–1.02)	0.647	1.29 (0.97–1.71)	0.073	1.42 (0.64–1.12)	0.017
Waist*	0.92 (0.69–1.23)	0.568	1.23 (0.93–1.65)	0.14	0.99 (0.67–1.48)	0.99	1.23 (0.87–1.37)	0.242	1.16 (0.81–1.67)	0.41
SBP (≤130 versus >130 mmHg)	**0.60** **(0.46–0.79)**	**<0.001**	1.09 (0.84–1.42)	0.493	1 (0.99–1.02)	0.274	**1.47** **(1.08–2)**	**0.014**	**1.47** **(1.07–2.02)**	**<0.001**
DBP (≤80 versus >80 mmHg)	0.81 (0.63–1.04)	0.102	1.19 (0.93–1.53)	0.166	0.98 (0.97–1.00)	0.146	**1.5** **(1.14–2.01)**	**0.005**	1.2 (0.87–1.57)	0.29
FPG (≤110 versus >110 mg/dL)	1.07 (0.81–1.41)	0.642	0.94 (0.71–1.24)	0.659	1.03 (0.68–1.55)	0.889	0.86 (0.64–1.2)	0.368	1.21 (0.88–1.68)	0.241
PPG (≤140 versus >140 mg/dL)	1.18 (0.85–1.63)	0.322	1.01 (0.73–1.4)	0.948	1.14 (0.7–1.84)	0.607	0.92 (0.65–1.3)	0.646	0.89 (0.62–1.28)	0.53
Neuro (no versus yes)	0.78 (0.59–1.04)	0.093	1.24 (0.94–1.63)	0.132	1.17 (0.77–1.77)	0.463	**1.56** **(1.13–2.18)**	**0.008**	**1.35** **(0.97–1.87)**	**0.07**
Nephro (no versus yes)	0.98 (0.76–1.26)	0.878	**1.43** **(1.12–1.83)**	**0.004**	—	—	1.08 (0.82–1.43)	0.57	**1.42** **(1.08–1.88)**	**0.014**
Retino (no versus yes)	1.04 (0.87–1.24)	0.662	**1.24** **(1.04–1.47)**	**0.017**	—	—	**1.39** **(1.15–1.68)**	**<0.001**	**1.19** **(0.98–1.4)**	**0.069**
CAD (no versus yes)	1.12 (0.79–1.59)	0.507	0.84 (0.59–1.12)	0.324	**2.52** **(1.44–4.41)**	**<0.001**	—	—	**1.89** **(1.32–2.73)**	**<0.001**
CVA (no versus yes)	1.55 (0.76–3.19)	0.23	0.55 (0.27–1.13)	0.106	1.46 (0.59–3.6)	0.407	1.53 (0.75–3.16)	0.246	1.46 (0.69–3.05)	0.316
DF (no versus yes)rs)	**0.81** **(0.65–1.009)**	**0.061**	1.18 (0.95–1.47)	0.14	1.15 (0.89–1.49)	0.279	0.83 (0.63–1.09)	0.194	1.21 (0.97–1.49)	0.088
Hb_A1C_ (≤7 versus >7%.)	1.006 (0.99–1.02)	0.334	0.99 (0.98–1)	0.548	—	—	0.98 (0.93–1.03)	0.409	0.99 (0.98–1.01)	0.633
Scr (≤1.2 versus >1.2%)	**0.84** **(0.71–0.99)**	**0.039**	1.09 (0.94–1.28)	0.24	0.97 (0.77–1.22)	0.816	**1.2** **(1.04–1.42)**	**0.016**	**1.52** **(1.25–1.85)**	**<0.001**
